# Regulation of CD4^+^CD8^−^CD25^+^ and CD4^+^CD8^+^CD25^+^ T cells by gut microbiota in chicken

**DOI:** 10.1038/s41598-018-26763-0

**Published:** 2018-06-05

**Authors:** In Kyu Lee, Min Jeong Gu, Kwang Hyun Ko, Suhan Bae, Girak Kim, Gwi-Deuk Jin, Eun Bae Kim, Young-Yun Kong, Tae Sub Park, Byung-Chul Park, Hyun Jung Jung, Seung Hyun Han, Cheol-Heui Yun

**Affiliations:** 1 Department of Agricultural Biotechnology and Research Institute for Agriculture and Life Sciences, Seoul National University, Seoul, 08826 Republic of Korea; 2 Department of Animal Life Science, College of Animal Life Science, Kangwon National University, Chuncheon, 24341 Republic of Korea; 3 School of Biological Sciences, Seoul National University, Seoul, 08826 Republic of Korea; 4 Graduate School of International Agricultural Technology and Institute of Green Bio Science Technology, Seoul National University, Pyeongchang, 25354 Republic of Korea; 5 National Institute of Animal Science, Wanju-gun, 55365 Republic of Korea; 6 Department of Oral Microbiology and Immunology, School of Dentistry, Seoul National University, Seoul, 08826 Republic of Korea; 7 Center for Food Bioconvergence, Seoul National University, Seoul, 08826 Republic of Korea

## Abstract

The gut microbiota in chicken has long been studied, mostly from the perspective of growth performance. However, there are some immunological studies regarding gut homeostasis in chicken. Although CD4^+^CD25^+^ T cells are reported to act as regulatory T cells (Tregs) in chicken, there have been no studies showing the relationship between gut microbiota and Tregs. Therefore, we established a model for ‘antibiotics (ABX)-treated chickens’ through administration of an antibiotic cocktail consisting of ampicillin, gentamycin, neomycin, metronidazole, and vancomycin in water for 7 days. CD4^+^CD8^−^CD25^+^ and CD4^+^CD8^+^CD25^+^ T cells in cecal tonsils were significantly decreased in this model. Gram-positive bacteria, especially Clostridia, was responsible for the changes in CD4^+^CD8^−^CD25^+^ or CD4^+^CD8^+^CD25^+^ T cells in cecal tonsils. Feeding ABX-treated chickens with acetate recovered CD4^+^CD8^−^CD25^+^ and CD4^+^CD8^+^CD25^+^ T cells in cecal tonsils. GPR43, a receptor for acetate, was highly expressed in CD4^+^CD8^−^CD25^+^ T cells. In conclusion, our study demonstrated that the gut microbiota can regulate the population of CD4^+^CD8^−^CD25^+^ and CD4^+^CD8^+^CD25^+^ T cells, and that acetate is responsible for the induction of CD4^+^CD8^−^CD25^+^ T cells in cecal tonsils via GPR43.

## Introduction

Tregs are a subtype of CD4^+^ T cell that are known to play an important role in maintaining gut immune homeostasis because the gastrointestinal tract is constantly exposed to microbial antigens with potential to induce inflammation^1^. In mouse and human, Foxp3 is the master transcription factor for Tregs^2,3^. Common surface molecules and cytokines used as markers for Tregs are CD25 (IL-2 receptor α), and IL-10 and TGF-β, respectively^4^. Non-Foxp3 Tregs, also called Tr1 cells^5^, which are induced by chronic activation of CD4^+^ T cells with antigen and IL-10^3^, have been reported. Although the master transcription factor for Tr1 cells is unknown, cytokine profiles for these cells are suggested to be IL-10^+^, TGF-β^+^, interferon (IFN)-γ^+^, IL-5^+^, IL-4^−^, and IL-2^low/− ^^3,6^. CD4^+^CD25^+^ T cells in chicken have been reported as Tregs^7,8^. Although *Foxp3* orthologue gene has not been identified in chickens yet^9^, there is a report for the existence of an avian *Foxp3* gene^10^.

A germ-free mouse model has been a critical tool for research on immune homeostasis in mucosal tissues and peripheral organs for decades^11–13^. Gut immune balance is the result of interactions among various immune cells including Tregs, Th17 cells, IgA-secreting B cells, and innate immune cells^13^. In indigenous germ-free mice, peripheral Tregs (pTregs) are scarce in the lamina propria of the intestine^14,15^. Antibiotic cocktail (ABX)-treated mice closely resemble indigenous germ-free mice in terms of immunological changes^16–18^. The presence of intestinal Th17 cells is dramatically reduced in ABX-treated mice^19^. Although Foxp3^+^ Tregs are still detectable, they are significantly decreased in colonic lamina propria^14^. To the best of our knowledge, there is no report on immunological research in ABX-treated chicken model.

Gut microbiota of chicken is dominated by the Firmicutes, and followed by others including Actinobacteria, Bacteroidetes and Proteobacteria^20^. Ceca are a part of hindgut with the highest density of microbiota and the fermentation of non-digestible carbohydrate^21^. Major cecal microbiota has been reported as Firmicutes genus followed by Lactobacillus and *Ruminococcus*^22^ and Clostridiaceae, Lachnospiraceae and Ruminococcaceae^23^. In other report, Enterococcaceae, Enterobacteriaceae and Bacteroidaceae are abundant in the cecal microbiota^24^. The functional role of short chain fatty acids (SCFAs) in chicken has been reported for preventing pathogens together with boosting weight gain^25,26^. Acidic environment (pH 5.5–6) in ceca of chicken could be caused by SCFAs, which are composed of acetate (55–75 mM), butyrate (15–25 mM) and propionate (5–10 mM)^27–29^, that consequently inhibit the increase of acid-sensitive pathogenic bacteria including Enterobacteriaceae^25^. However, immunological functions of SCFAs have not been solely studied in chickens yet.

In the present study, we established a model for studying gut immune homeostasis in chickens treated with ABX. The main goals of the study were (1) to examine the changes in populations and function of immune cells in ABX-treated chickens and (2) to identify the factors regulating gut immune homeostasis.

## Results

### ABX treatment reduces gut microbiota in chickens

We examined how ABX impacted gut microbiota in chickens treated with various concentrations of ABX containing ampicillin, gentamycin, metronidazole, neomycin, and vancomycin (Table S1) provided in the drinking water^30^ *ad libitum* for 7 days. Colonies were not observed from cecal contents of chickens treated with ABX (1:10) (Fig. S1). ABX treated chickens will, hereafter, refer to those who received ABX at a 1:10 dilution.

### Physiological changes occur on chickens by ABX treatment

No significant differences in body weight or lengths of distinct regions of small intestine (duodenum and jejunum + ileum) and large intestine (Fig. S2A,B) were observed. The amount of glucocorticoid in serum, as a stress marker, was not changed (Fig. S2C). Furthermore, the weights of major organs including spleen, bursa, and liver were not altered (Fig. S2D). It was noting that cecal length/weight was increased (Fig. S2E). Water consumption after ABX treatment did not make any differences between control chickens (Con) and ABX-treated chickens (ABX) (data not shown). Taken together, we observed that ABX treatment in chickens induced slightly bigger ceca, but not other major immune organs.

### CD4^+^CD8^−^CD25^+^ and CD4^+^CD8^+^CD25^+^ T cells in cecal tonsils are changed in ABX-treated chickens

**Figure 1:**
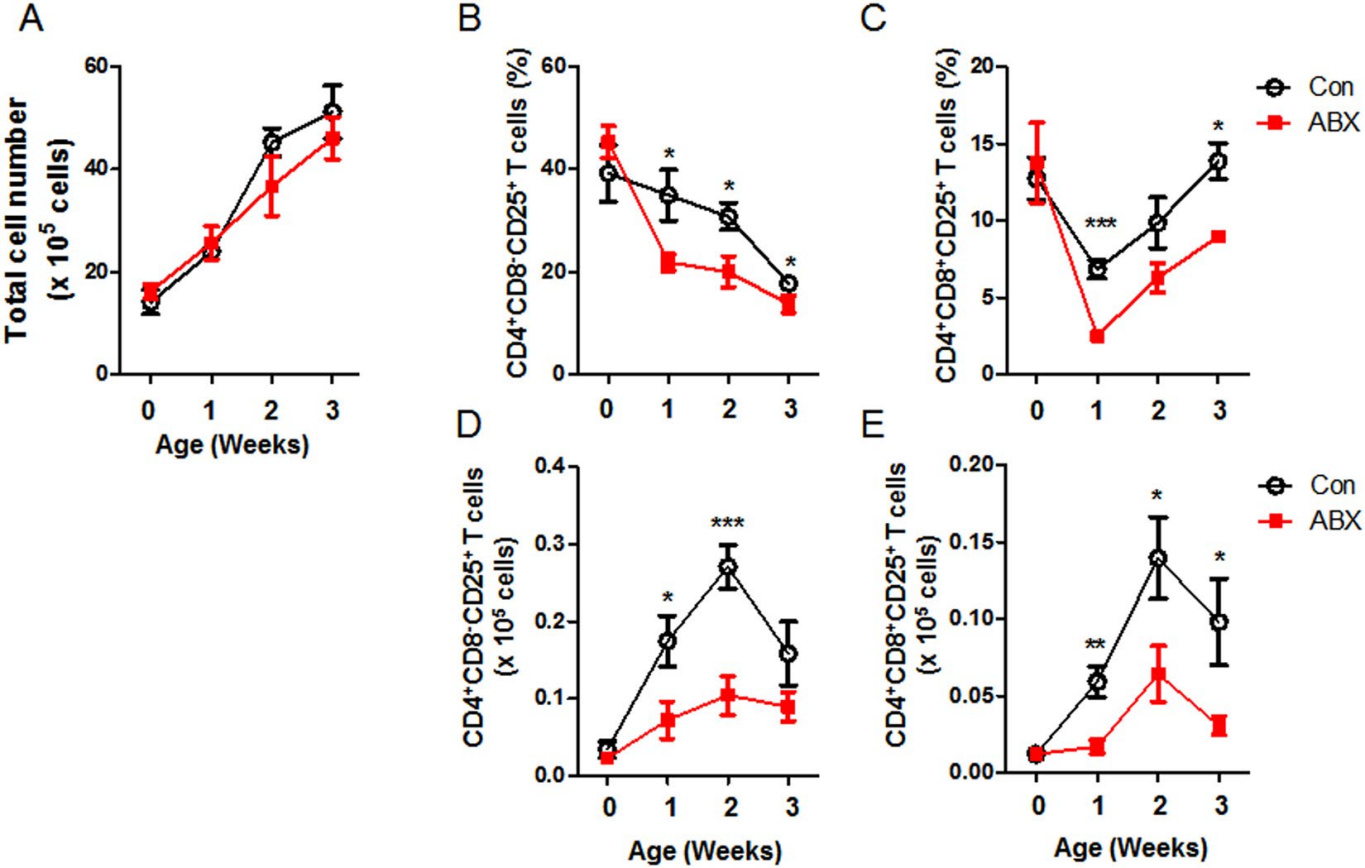
Numbers of CD4^+^CD8^−^CD25^+^ and CD4^+^CD8^+^CD25^+^ T cells were reduced in cecal tonsils of ABX-treated chickens. Chickens were given water containing antibiotics at hatching for 3 weeks, and cecal tonsils were taken. Single cells from cecal tonsils were stained with anti-chicken TCRγδ, CD3, CD4, CD8α, and CD25 antibodies. The cells were pre-gated for CD3^+^γδTCR^−^ cells. Changes in (**A**) the total number of cells, the percentages of (**B**) CD4^+^CD8^−^CD25^+^ and (**C**) CD4^+^CD8^+^CD25^+^ T cells, and the absolute numbers of (**D**) CD4^+^CD8^−^CD25^+^ and (**E**) CD4^+^CD8^+^CD25^+^ T cells are shown. (**A**–**E**) Data were obtained from six chickens in each group and presented as mean ± SD. Asterisks indicate significant differences between Con and ABX. Data are representative of three independent experiments. ^*^P < 0.05, ^**^P < 0.01, ^***^P < 0.001.

CD4^+^CD8^+^ T cells were previously reported in chicken^31^. Indeed, we confirmed that CD4^+^ T cells could be distinguished into four subtypes using antibodies to CD4, CD8, and CD25 (Fig. S3). To examine the percentage and absolute number of CD4^+^ subtype T cells in cecal tonsils, flow cytometric analysis was performed after staining with anti-chicken TCRγδ, CD3, CD4, CD8α, and CD25 antibodies. CD3^+^γδTCR^−^ cells were pre-gated, and then CD4^+^ T cells were divided into CD4^+^CD8^−^ and CD4^+^CD8^+^ T cells. Finally, CD25^+^ cells were analyzed (Fig. 1). Total cell number of cecal tonsils showed no significant changes in ABX-treated chickens compared with control chickens (Fig. 1A). Furthermore, there were no changes in αβ T cells (Fig. S4A,D), CD4^+^CD8^−^ (Fig. S4B,E), or CD4^+^CD8^+^ (Fig. S4C,F) T cells. Interestingly, the amounts of CD4^+^CD8^−^CD25^+^ (Fig. 1B,D) and CD4^+^CD8^+^CD25^+^ (Fig. 1C,E) T cells from cecal tonsils were significantly reduced in ABX-treated chickens compared with control, whereas no significant changes in CD4^+^CD8^−^CD25^+^ and CD4^+^CD8^+^CD25^+^ T cells were observed in the spleen (Fig. S5).

### IL-10 and IFN-γ levels are decreased in cecal CD4^+^CD8^−^CD25^+^ T cells of ABX-treated chickens

**Figure 2:**
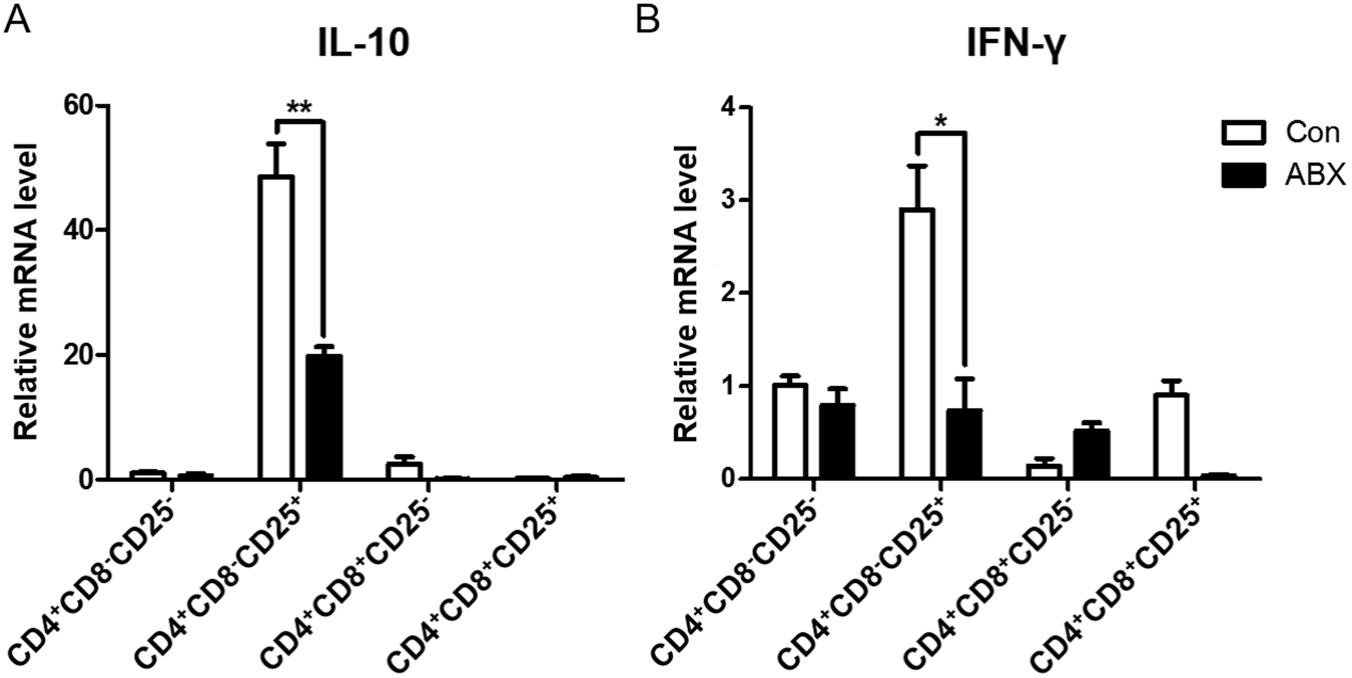
Expression of *IL-10* and *IFN-γ* mRNA among CD4^+^ T cell subsets in cecal tonsils of ABX-treated chickens. Chickens were given water containing antibiotics at hatching for 7 days, and cecal tonsils were taken. Single cells from cecal tonsils were stained with anti-chicken CD4, CD8α, and CD25 antibodies. Each subset of CD4^+^ T cells was sorted using an ARIA II FACS sorter. The mRNA was extracted from each subset, and the expression levels of (**A**) *IL-10* and (**B**) *IFN-*γ were determined by RT-qPCR. Data were obtained from three chickens in each group and presented as the mean ± SD. Asterisks indicate significant differences between Con and ABX. ^*^P < 0.05 and ^**^P < 0.01.

We examined whether the reduction of gut microbiota affects the expression of cytokines in a subset of CD4^+^ T cells. Interestingly, mRNA expression of both *IL-10* (Fig. 2A) and *IFN-*γ (Fig. 2B) in CD4^+^CD8^−^CD25^+^ T cells from cecal tonsils was significantly reduced in ABX-treated chickens.

### Antibiotics do not induce direct toxicity and downregulation of CD25

To examine the possibility of direct reduction of these T cells by antibiotics, we performed an *in vitro* experiment in which splenocytes were treated with pre-determined (data not shown) amounts of each antibiotic or a combination of antibiotics for 24 h. There were no significant differences in the cell number (Fig. S6A) or proportion (Fig. S6B) of these cells compared with control. These results suggested that the reduction of CD4^+^CD8^−^CD25^+^ T cells in ABX-treated chickens was not directly mediated by the antibiotics.

### Peripheral CD5^hi^ populations of CD4^+^CD8^−^CD25^+^ and CD4^+^CD8^+^CD25^+^ T cells are altered in cecal tonsils of ABX-treated chickens

It has been reported that CD5^hi^CD4^+^CD25^−^Foxp3^−^ T cells preferentially develop into peripheral Foxp3^+^ Tregs in mice^32^. We examined CD5 expression of CD4^+^CD8^−^CD25^+^ and CD4^+^CD8^+^CD25^+^ T cells in peripheral organs of ABX-treated chickens. The results showed that CD5 expression was decreased in both CD4^+^CD8^−^CD25^+^ and CD4^+^CD8^+^CD25^+^ T cells in cecal tonsils of ABX-treated chickens (Fig. S7).

CD4^+^CD25^+^ T cells preferentially migrate from thymus to cecal tonsils^33^; therefore, the reduction of CD4^+^CD8^−^CD25^+^ and CD4^+^CD8^+^CD25^+^ T cells in cecal tonsils could be the result of reduced migration from the thymus. In chicken thymus, CD4^+^CD8^+^ T cells are the major population of CD4^+^ T cells (Fig. S8A). There was no change in CD5 expression on CD4^+^CD8^+^CD25^+^ T cells in the thymus of ABX-treated chickens compared with control chickens (Fig. S8). Taken together, these findings indicate that the reductions of CD4^+^CD8^−^CD25^+^ and CD4^+^CD8^+^CD25^+^ T cells in cecal tonsils of ABX-treated chickens were not due to low emigration of these cells from the thymus.

### CD4^+^CD8^−^CD25^+^ and CD4^+^CD8^+^CD25^+^ T cells are recovered in ABX-treated chickens after co-housing with control chickens

**Figure 3:**
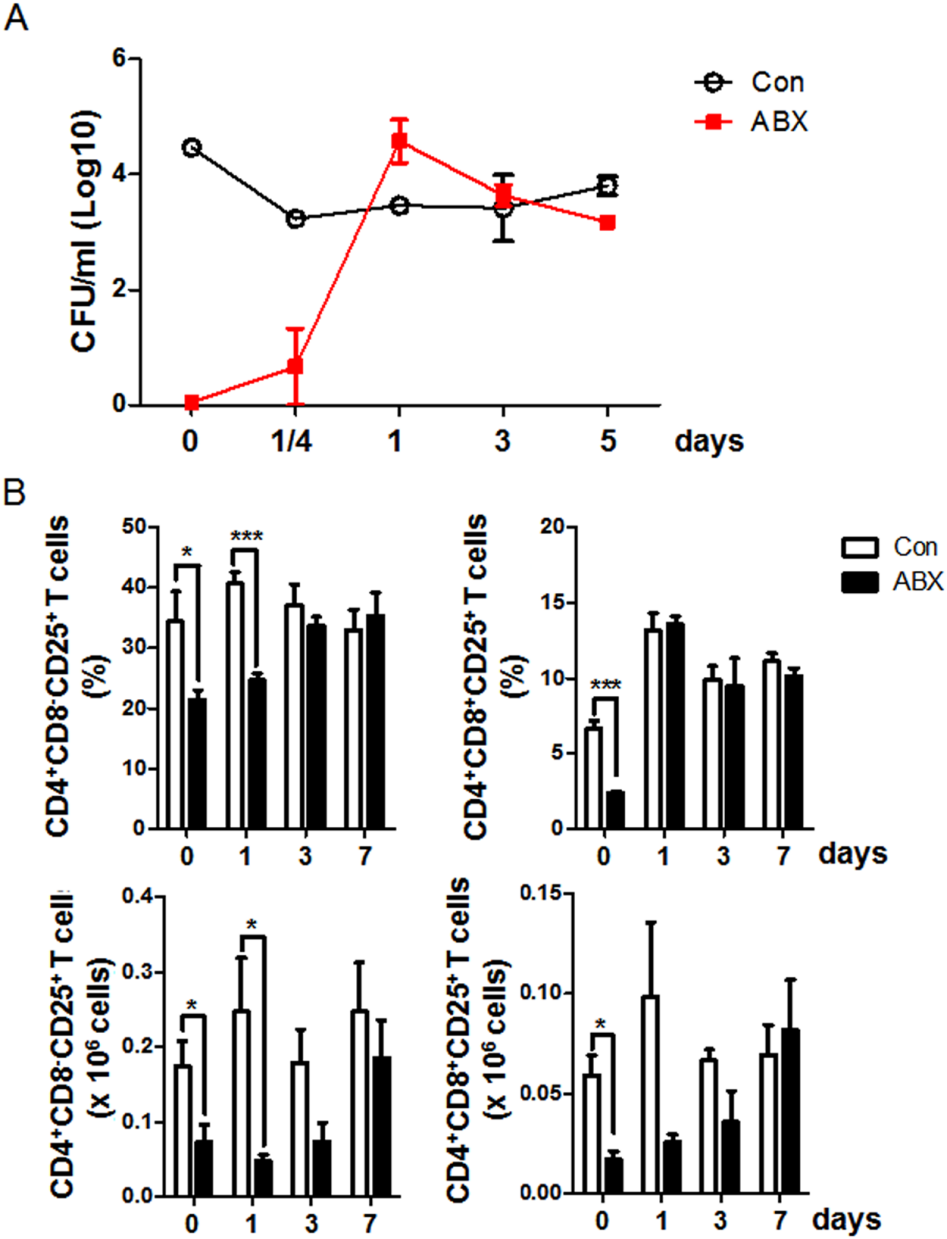
Changes in microbial number (colony forming units; CFU), CD4^+^CD8^−^CD25^+^, and CD4^+^CD8^+^CD25^+^ T cells in cecal tonsils of ABX-treated chickens after co-housing with control chickens. Chickens were treated with ABX at hatching for 7 days and then co-housed with control chickens for 7 days. (**A**) CFU was measured from cecal contents (1 mg/ml) at 1/4 (6 hours), 1, 3, and 5 days after co-housing. (**B**) Proportions and numbers of CD4^+^CD8^−^CD25^+^ and CD4^+^CD8^+^CD25^+^ T cells in cecal tonsils were analyzed by flow cytometry after co-housing. Data were obtained from more than six chickens in each group and presented as the mean ± SD. Significant differences between Con and ABX are shown by asterisks, and data are representative of three independent experiments. ^*^P < 0.05 and ^***^P < 0.001.

CD4^+^CD8^−^CD25^+^ and CD4^+^CD8^+^CD25^+^ T cells were significantly reduced in ABX-treated chickens (Fig. 1). We examined whether the reconstitution of gut microbiota is concordant with recovery of CD4^+^CD8^−^CD25^+^ and CD4^+^CD8^+^CD25^+^ T cells in ABX-treated chickens after co-housing with control chickens. Bacterial colonies were observed as early as 1/4 day (6 hours) post co-housing and reached a similar level to the control at 1 day post co-housing (Fig. 3A). The phylogenetic clusters of gut microbiota from control and ABX-treated chickens were separated at 5 days after ABX treatment and merged at 5 days post co-housing (Fig. S9), indicating that it took about 5 days for ABX to effectively induce gut microbiome changes. Interestingly, the numbers of CD4^+^CD8^−^CD25^+^ and CD4^+^CD8^+^CD25^+^ T cells gradually increased to similar levels as the control at 7 days post co-housing (Fig. 3B), suggesting that gut microbiota could influence the number and function of CD4^+^CD8^−^CD25^+^ and CD4^+^CD8^+^CD25^+^ T cells.

### Gram-positive bacteria are critical for induction of CD4^+^CD8^−^CD25^+^ and CD4^+^CD8^+^CD25^+^ T cells

Next, we examined whether Gram-positive or Gram-negative bacteria influenced the changes in CD4^+^CD8^−^CD25^+^ and CD4^+^CD8^+^CD25^+^ T cells. Selective deletion of bacteria was performed using vancomycin (Van) to eliminate Gram-positive bacteria and polymyxin B (PolyB) to reduce Gram-negative bacteria^14^. The total CFU of Van- or PolyB-treated chickens was slightly higher than that of the control (Fig. S10A). PolyB completely eliminated Gram-negative bacteria. Van reduced Gram-positive bacteria from 33% to 7% (Fig. S10B). Surprisingly, CD4^+^CD8^−^CD25^+^ and CD4^+^CD8^+^CD25^+^ T cells were significantly decreased by treatment with Van, but not with PolyB (Fig. S10C). To confirm the effect of Van, we examined another group, ABX without vancomycin (Without Van), and found no significant differences (Fig. S10D), indicating that the change was indeed caused by loss of Gram-positive bacteria. Taken together, these data suggest that Gram-positive bacteria play a critical role in the induction of CD4^+^CD8^−^CD25^+^ and CD4^+^CD8^+^CD25^+^ T cells in cecal tonsils.

**Figure 4:**
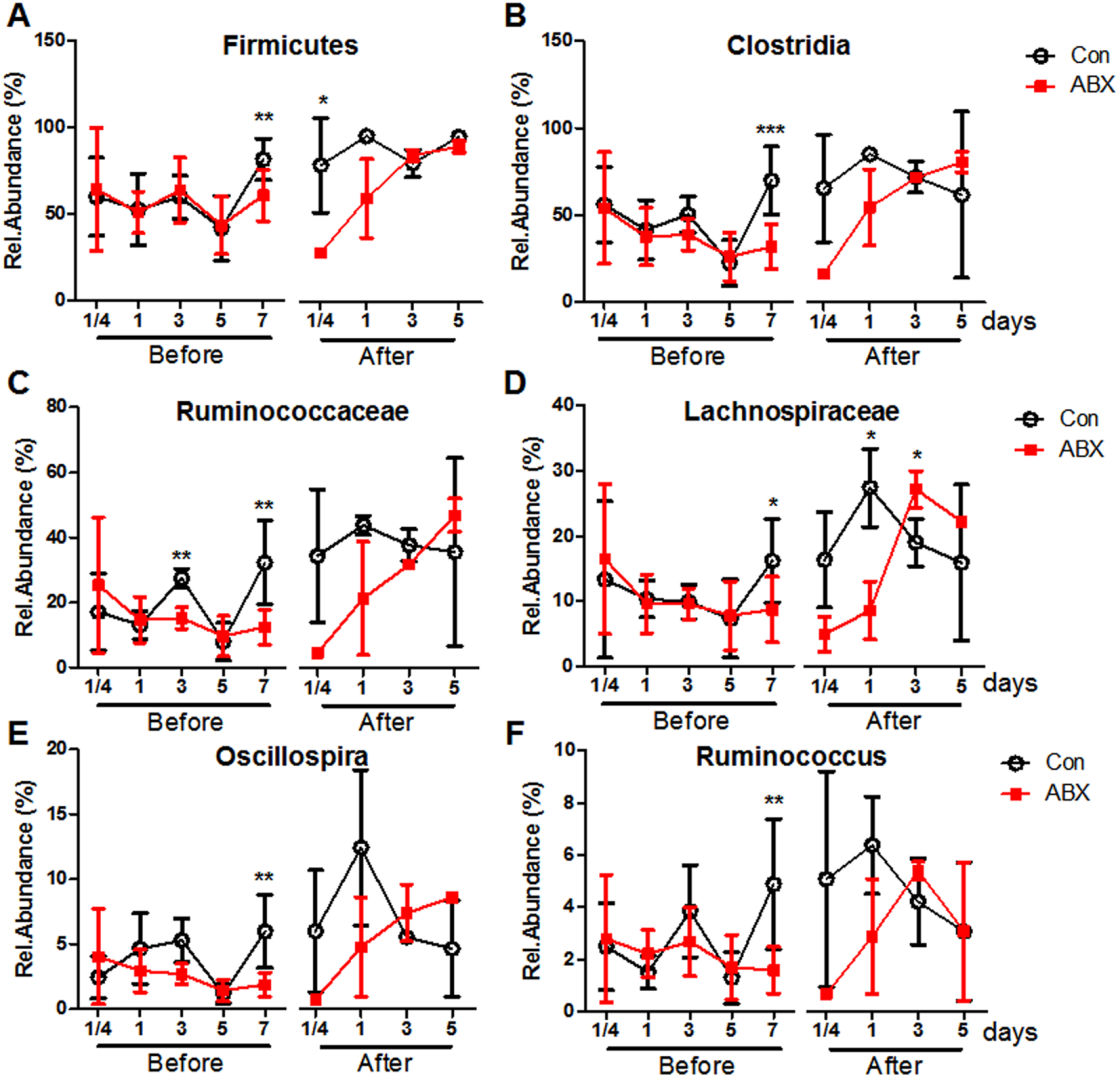
Changes in Clostridia in cecal contents from ABX-treated chickens after co-housing with control chickens. Chickens were treated with ABX at hatching for 7 days and then co-housed with control chickens for 7 days. 16S rRNA sequencing was performed to determine the relative abundance of (**A**) Phylum, (**B**) Class, (**C**,**D**) Family, and (**E**,**F**) Genus in cecal contents at 1/4 (6 hours), 1, 3 and 5 days before co-housing (Before), and 1/4 (6 hours), 1, 3, and 5 days after co-housing (After). Data were obtained from more than four chickens in each group and presented as the mean ± SD. Significant differences between Con and ABX are shown with asterisks. ^*^P < 0.05, ^**^P < 0.01, and ^***^P < 0.001.

Phylogenetic analysis showed that the abundance of Firmicutes (Phylum) (Fig. 4A), Clostridia (Class) (Fig. 4B), Ruminococcaceae, and Lachnospiraceae (Family) (Fig. 4C,D) were decreased at 7 days post ABX treatment. Two species belonging to Clostridia, *Ruminococcus* and *Oscillospira* (Genus), were reduced by ABX treatment (Fig. 4E,F). Interestingly, the abundance of Proteobacteria (Phylum) was increased by ABX treatment (Fig. S11). These results demonstrated that Gram-positive bacteria, especially *Clostridia*, were the most effective in the induction of CD4^+^CD8^−^CD25^+^ and CD4^+^CD8^+^CD25^+^ T cells.

### Feeding with acetate rescues CD4^+^CD8^−^CD25^+^ and CD4^+^CD8^+^CD25^+^ T cells *in vivo*

**Figure 5:**
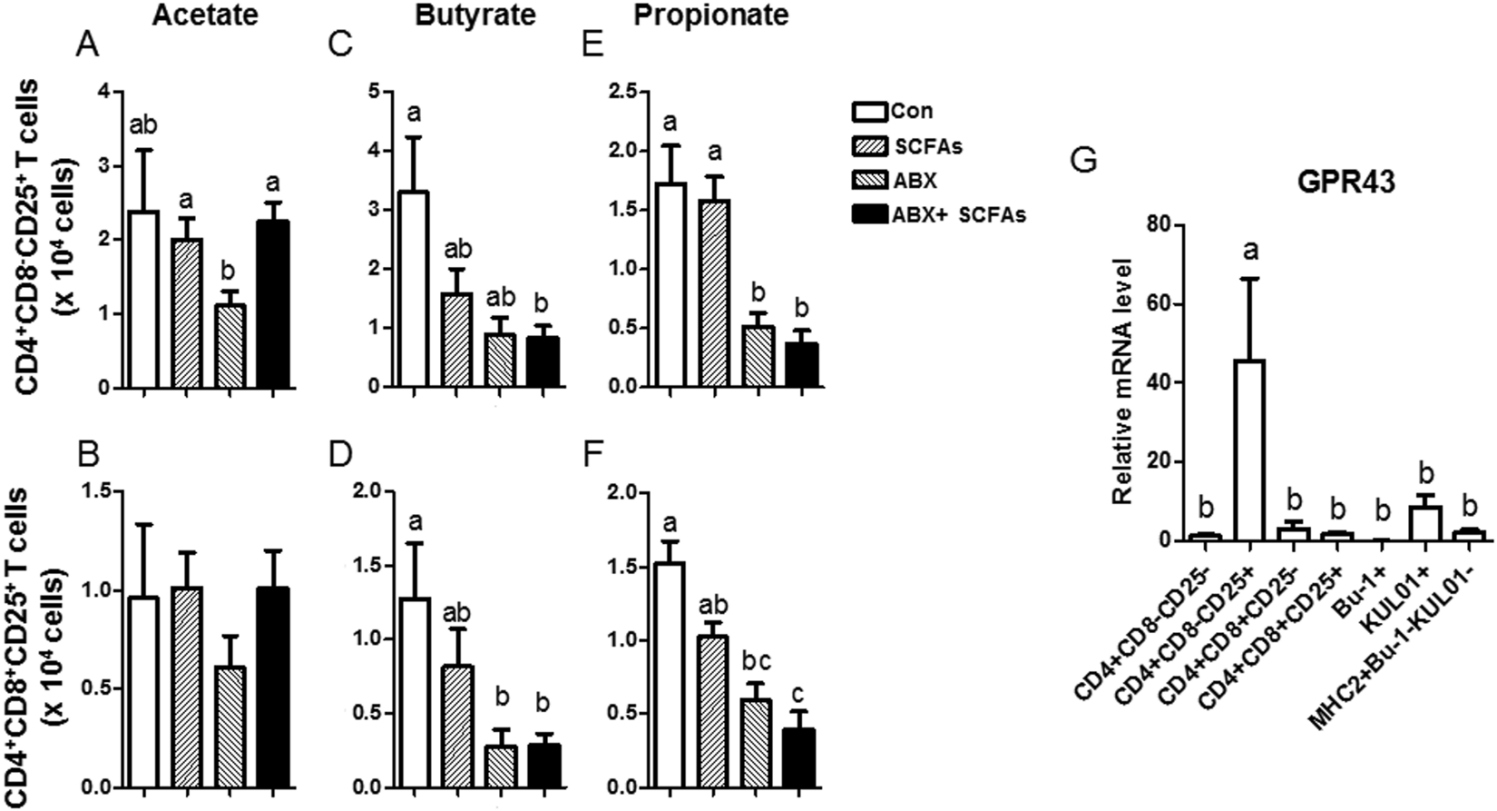
Changes in CD4^+^CD8^−^CD25^+^ and CD4^+^CD8^+^CD25^+^ T cells in chickens treated with acetate. SCFAs (acetate 50 mM, butyrate 30 mM, propionate 10 mM) and/or ABX in drinking water were administered to chickens at hatching for 7 days. The numbers of (**A**,**C**,**E**) CD4^+^CD8^−^CD25^+^ and (**B**,**D**,**F**) CD4^+^CD8^+^CD25^+^ T cells in cecal tonsils were calculated. (**G**) Subsets of CD4^+^ T cells, B cells (Bu-1^+^), and APCs (KUL01^+^, MHC class II (MHC2)^+^KUL01^−^Bu-1^−^) were sorted using an ARIA II FACS sorter. mRNA was extracted from each subset, and the expression level of GPR43 was determined by RT-qPCR. (**A**–**G**) Data were obtained from more than three chickens in each group and presented as the mean ± SD. Different characters indicate significant differences at *P* < 0.05. The figures are representative of three independent experiments.

It has been suggested that SCFAs are one of the factors that induce Tregs or Tr1 in mice^34^. We therefore examined whether SCFAs affect the population of CD4^+^CD8^−^CD25^+^ and CD4^+^CD8^+^CD25^+^ T cells in chickens. It was intriguing that ABX-treated chickens administered acetate recovered CD4^+^CD8^−^CD25^+^ T cells in cecal tonsils (Fig. 5A). CD4^+^CD8^+^CD25^+^ T cells also showed a tendency for recovery but with non-significant differences (Fig. 5B). The other SCFAs, butyrate and propionate, did not show such effects (Fig. 5C–F). GPR43 is known as a receptor for acetate^35^. *GPR43* mRNA expression in CD4^+^CD8^−^CD25^+^ T cells was significantly higher than in other immune cells (Fig. 5G) and acetic acid in ceca was reduced remarkably by ABX treatment (Fig. S12), which strongly suggests that the recovery of CD4^+^CD8^−^CD25^+^ T cells by acetate administration in ABX-treated chickens might be associated with high GPR43 expression on CD4^+^CD8^−^CD25^+^ T cells.

## Discussion

The purpose of the present study was to investigate the impact of gut microbiota on intestinal Tregs in chicken. The model was established to reduce gut microbiota in chickens by treatment with antibiotics, designated ABX-treated chickens. We demonstrated that the proportions and absolute numbers of CD4^+^CD8^−^CD25^+^ and CD4^+^CD8^+^CD25^+^ T cells were significantly diminished in cecal tonsils of chickens after the reduction of gut microbiota. In contrast, there was no change in CD4^+^CD8^+^CD25^+^ T cells in the thymus.

Expression of IL-10 and IFN-γ on CD4^+^CD8^−^CD25^+^ and CD4^+^CD8^+^CD25^+^ T cells were significantly decreased by the reduction of gut microbiota. Gram-positive bacteria, especially *Clostridia*, appeared to be responsible for the recovery of CD4^+^CD8^−^CD25^+^ and CD4^+^CD8^+^CD25^+^ T cells. Furthermore, CD4^+^CD8^−^CD25^+^ T cells in cecal tonsils of ABX-treated chickens were induced by acetate administration. Furthermore, GPR43 was highly expressed in CD4^+^CD8^−^CD25^+^ T cells.

We demonstrated the high expression of IL-10 and IFN-γ in CD4^+^CD8^−^CD25^+^ and CD4^+^CD8^+^CD25^+^ T cells in cecal tonsils from ABX-treated chickens. We postulated that CD4^+^CD8^−^CD25^+^ and CD4^+^CD8^+^CD25^+^ T cells might resemble Tr1 cells, known as non-Foxp3 Tregs in human and mouse^3^, since there is no Foxp3 gene in chicken^9^. Furthermore, CD4^+^CD8^−^CD25^+^ T cells expressed IL-10 and IFN-γ (Fig. 2). Previously, Chicken CD4^+^CD25^+^ T cells are shown to express high levels of IL-10 and acted as Tregs^7^. Indeed, it has been demonstrated that Tr1 cells produce IL-10 and IFN-γ at much higher levels than Foxp^+^ Tregs in mouse^36^. We examined transcription factors associated with Tr1 cells, namely cellular homologs of the avian virus oncogene musculoaponeurotic fibrosarcoma (Maf) and aryl hydrocarbon receptor (Ahr)^5^. No significant differences in Ahr mRNA level among CD4^+^ subtype T cells were found (Fig. S13); however, the expression of Maf was high in CD4^+^CD8^−^ subtype T cells. It has been suggested that the kinetics of both Maf and Ahr are increased coincidently with Tr1 induction and expression of the cytokines TGF-β and IL-27^37^. The precise molecular mechanisms of Maf and Ahr functions in CD4^+^CD8^−^CD25^+^ and CD4^+^CD8^+^CD25^+^ T cells in chickens should be further investigated.

There are few, if any, studies on the function of CD4^+^CD8^+^ T cells in chicken. Peripheral CD4^+^CD8^+^ T cells, analyzed in the current study, are referred to as CD4^+^CD8a^+^ (double positive; DP) T cells in human and other chicken studies. DP T cells represent a very small population (<3%) in the blood of healthy people^38^. DP T cells express lower levels of CD8α than CD8^+^ cytotoxic T cells^39^. It has been shown that human intestinal DP T cells express IL-10 and IFN-γ, but not Foxp3^40^. Human intestinal DP T cells are known to suppress proliferation of CD4^+^ T cells^40^. DP T cells are significantly decreased in the lamina propria of patients with inflammatory bowel disease^40^. Intestinal DP T cells express IL-10 or IFN-γ specifically when they are stimulated with *Faecalibacterium prausnitzii*, a *Clostridium* cluster IV strain^40^. In mice, DP intraepithelial lymphocytes (IELs) are known to produce IL-10 and prevent type 1 helper T (Th1) cell-induced intestinal inflammation in a GATA3-dependent manner^40^. In chicken, DP T cells are observed in the peripheral blood (20–40%), spleen (10–20%), and intestinal epithelium (5–10%)^31^, but the functions of DP T cells have not yet been studied in detail.

The reductions of CD4^+^CD8^−^CD25^+^ and CD4^+^CD8^+^CD25^+^ T cells in cecal tonsils of ABX-treated chickens could be affected by low levels of SCFAs. In mouse studies, the induction and function of Tregs were affected by SCFAs^34,41,42^ including acetate, propionate, and butyrate^43^, which are generated especially by Firmicutes and Bacteroidetes after fermentation of undigested carbohydrates^1^. Activation of GPR43 using SCFAs promotes the number and function of IL-10^+^Foxp3^+^ Tregs, and propionate directly increases Foxp3 expression and IL-10 production^34^. Indeed, both butyrate and propionate are known to induce the differentiation of Foxp3^+^ Tregs^34^. Interestingly, however, only acetate, but not propionate or butyrate, induced CD4^+^CD8^−^CD25^+^ and CD4^+^CD8^+^CD25^+^ T cells in cecal tonsils in the present study. There are a few possible reasons for this. First, propionate induces colonic Foxp3^+^ Tregs via GPR43 *in vivo*^34^, whereas there is no evidence of the induction of Tr1 cells. Second, butyrate stimulates the secretion of IL-10 and RA from dendritic cells (DCs) and macrophages via GPR109α expressed in DCs and macrophages, but not in T cells^44,45^, to induce Foxp3^+^ Treg and Tr1 cells^46^. However, the GPR109α gene does not exist in chicken^47,48^.

How CD4^+^CD8^−^CD25^+^ T cells were affected by acetate is not clear. Acetate can induce the differentiation of naïve T cells to Tr1 cells directly through a GPR43-independent pathway, whereas it acetylates p70 S6 kinase and activates ribosomal protein S6 (rS6) through HDAC inhibitor activity^49^. In contrast, another study suggested that SCFAs can directly suppress HDAC in a GPR43-dependent manner^34^. In addition, the role of GPR43 expression in the regulatory function of T cells has been controversial^34,35,50^.

CD4^+^CD25^+^ T cells are shown to preferentially migrate to cecal tonsils^33^. Therefore, another possibility for the reduction of CD4^+^CD8^−^CD25^+^ and CD4^+^CD8^+^CD25^+^ T cells in ABX-treated chickens might be reduced migration of these cells. However, there were no changes in CD4^+^CD8^+^CD25^+^ T cells in the thymus in ABX-treated chickens. Collectively, these findings indicate that migration is unlikely to be the mechanism for the reduction of CD4^+^CD8^−^CD25^+^ and CD4^+^CD8^+^CD25^+^ T cells in ABX-treated chickens.

The present study demonstrated that CD4^+^CD8^−^CD25^+^ and CD4^+^CD8^+^CD25^+^ T cells were affected by Gram-positive bacteria, in particular Clostridia *Ruminococcus*, and *Oscillospira*. *Ruminococcus albus* ferments carbohydrate to acetate *in vitro*^51,52^. *Ruminococcus* is a member of *Clostridium* cluster XIVa^53^, which produces abundant acetate and a lesser amount of butyrate^54^. *Oscillospira* shows a positive correlation with acetate^55^. Conversely, both *Ruminococcus* and *Oscillospira* promote pathogenesis of type 1 diabetes, which is prevented by Tregs. Unfortunately, there is no study on whether these kinds of bacteria induce Tr1. The probiotics *Bifidobacterium breve* and *B. longum* induce colonic Tr1 via CD103^+^ DCs that ameliorate severe intestinal inflammation^56^. *Clostridium* cluster IV and XIVa produce abundant acetate with a small amount of butyrate^54^. Therefore, Clostridia probably affect intestinal CD4^+^CD8^−^CD25^+^ and CD4^+^CD8^+^CD25^+^ T cells in chickens.

It has been suggested that Firmicutes and Bacteroidetes are core microbiota in healthy human^57^. Firmicutes is also the major phyla in chicken^58^. In chickens treated with ABX for 7 days, Firmicutes was decreased and Proteobacteria was increased significantly. It is probable that core microbiota of chickens treated with ABX is collapsed leading to dysbiosis.

Antibiotics seemingly affect not only the population of microbiota, but also metabolism in the host. Although the precise action mode of antibiotics in promoting growth of domestic animals is still unclear, it is widely accepted that antibiotics modulate the gut microbiome and its products, such as short-chain fatty acids^59,60^, causing changes in the magnitude of host immunity. Of course, suppression of enteric pathogens, for example, *Escherichia coli*, *Salmonella ssp*., and *Clostridium perfringens*, would be an extra benefit for healthy intestinal epithelium^61–63^. However, how antibiotics specifically target those enteric pathogens and not common microbes is yet to be determined and difficult to explain.

Collectively, the results of the present study suggest that the gut microbiota regulates both the population and function of CD4^+^CD8^−^CD25^+^ and CD4^+^CD8^+^CD25^+^ T cells in cecal tonsils, and acetate can play an important role in gut immune homeostasis. It is likely that acetate produced by Gram-positive bacteria, especially *Oscillospira* and *Ruminococcus*, could be used as probiotics to improve gut health. Furthermore, the ABX-treated chicken model could be used for future studies on the relationship between gut homeostasis and microbes, including probiotics and synbiotics.

## Methods

### Experimental animals and animal care

All animal experimental procedures were approved by the Institutional Animal Care and Use Committee of Seoul National University (IACUC No., SNU-150327-2). All White Leghorn chickens were maintained and handled according to a standard management program at the University Animal Farm.

### Determination of ABX dilution factor

For the antibiotics-treated group, chickens were treated at hatching with various concentrations of antibiotics in drinking water *ad libitum* for 7 days. We defined dilution factor (DF) 1 as an antibiotic cocktail containing ampicillin, gentamycin, neomycin (all from Sigma-Aldrich, St. Louis, MO), and metronidazole (Abcam, Cambridge, MA) at 1 mg/ml each and vancomycin (Sigma-Aldrich) at 0.5 mg/ml. DFs of 1:1, 1:2, 1:10, and 1:20 were tested. For further experiments, ABX-treated chickens were treated with 1:10 diluted antibiotics for 1 or 3 weeks and sacrificed at finishing ABX treatment.

### Measurement of colony forming unit (CFU)

Cecal contents from chickens treated with ABX for 7 days were dissolved in PBS to adjust the concentration to 1 mg/ml. Dissolved cecal contents from the control chickens were diluted 100–1,000 fold with PBS, whereas those from the ABX-treated chickens were used without dilution. All dissolved cecal contents were spread on Brain Heart Infusion (BHI) agar media (BD Biosciences, San Jose, CA) and incubated at 37 °C for 12 hours. The number of CFU was determined by counting the number of colonies.

### Examination of physiological changes in ABX-treated chickens

Body weight changes were monitored in chickens every day for 7 days. At the end of the experiment, major immune organs (liver, spleen, and bursa) were harvested, briefly semi-dried by tapping on a paper towel, and the weight was examined. The length of intestine was segmented into jejunum (J), duodenum and ileum (D + I), Cecum (C), and large intestine (L) and measured on a millimeter scale. Blood samples from a wing vein were collected 7 days after ABX treatment. The amount of glucocorticoid in serum was measured by a chicken glucocorticoid ELISA kit (MyBioSource, San Diego, CA) according to the manufacturer’s specification. Absorbance was measured at 450 nm using an ELISA microplate reader (Molecular Device, Sunnyvale, CA), and the amount of glucocorticoid was calculated from the standard curve.

### Changes in the subtypes of CD4^+^ T cells after treatment with antibiotics *in vitro*

Spleens from 2- to 3-week-old chickens were harvested, and single cells were generated as described in the following section. Splenocytes (1 × 10^5^ cells/well) in a 96-well culture plate (Nunc, Roskilde, Denmark) were treated with 100 μg/ml of ampicillin (A), gentamycin (G), metronidazole (M), and neomycin (N) and 50 μg/ml of vancomycin (V) for 24 hours. Changes in CD4^+^ subtype T cells were analyzed by flow cytometry with anti-chicken CD4-FITC (clone CT-4), CD8α-PE (clone CT-8) (all from Southern Biotec), and CD25-Alexa Fluor^®^ 647 (clone 13504; AbD Serotec, Puchheim, Germany) antibodies. Total cell numbers were determined using an automatic cell counter TC10. The number of each CD4^+^ subtype of T cells and the proportion of CD4^+^ subtype T cells relative to total cell number was analyzed using FlowJo software.

### Conditional elimination of Gram-positive and Gram-negative bacteria

Chickens were treated at hatching for 7 days with vancomycin (100 μg/ml; Van) for the removal of Gram-positive bacteria or with polymyxin B (10 μg/ml; PolyB) for removal of Gram-negative bacteria. CFU of cecal contents (1 mg/ml) was measured. Gram staining was performed using a kit (BD Biosciences). Briefly, unknown bacterial samples from colonies were smeared in 10 μl distilled water onto the slide and then fixed by quickly passing through a flame 2-3 times. The samples were sequentially flooded with crystal violet solution for 1 minute, flooded with iodine solution for 1 minute, washed with decolorizer for 10 seconds, and flooded with safranin for 30 seconds, rinsing with tap water between each stage. Finally, samples were dried with absorbent paper and examined for Gram-positive or Gram-negative bacteria under a microscope.

### Flow cytometric analysis of immune cells

After washing, chunked spleen or longitudinally cut cecal tonsils were minced with the flat end of a 3-ml syringe plunger through a 40-μm cell strainer (BD Biosciences, San Jose, CA) into a 50-ml conical tube (SPL, Pocheon, Korea). To purify immune cells, red blood cells were lysed using ACK buffer (BD Biosciences) for 3 min at room temperature and then washed.

For examination of B cells and macrophages, anti-chicken MHC class II-FITC (clone 2G11), Monocyte/Macrophage-PE (clone KUL01), and Bu-1-Alexa Flour^®^ 647 (clone AV20) antibodies (all from Southern Biotec, Birmingham, AL) were used. To examine CD4^+^ subtypes of T cells, anti-chicken CD3-Percific Blue (clone CT-4), CD4-FITC, CD8α-SPRD (clone CT-8), TCRgd-PE (clone TCR1), CD5-biotin (clone 2-191) (all from Southern Biotec), and CD25-Alexa Fluor^®^ 647 (AbD Serotec) antibodies and Brilliant Violet 605 streptavidin (BioLegend, San Diego, CA) were used.

Data acquired by flow cytometry (FACS Canto II, BD Biosciences) were analyzed with FlowJo software (Tree Star, San Carlos, CA). Total cell number was determined by an automatic cell counter TC10 (Bio-Rad, Hercules, CA). The number and proportion of immune cells were calculated.

### Measurement of mRNA level using RT-qPCR

CD4^+^ subtypes of T cells (CD4^+^CD8^−/+^CD25^−/+^), B cells (Bu-1^+^), and APCs (KUL01^+^ as macrophages, MHC class II^+^Bu-1^−^KUL01^−^) were sorted using an ARIA II FACS sorter (BD Biosciences). Total RNA of each CD4^+^ subtype of T cells was extracted using a miRNeasy Micro Kit (QIAGEN, Hilden, Germany). SYBR Green PCR Master Mix was used according to the manufacturer’s specification (Applied Biosystems). Relative quantification of target genes was performed using the 2^−ΔΔCt^ method. Target gene expression was normalized to β-actin mRNA level. Primers for IL-10, IFN-γ, Ahr, Maf, G-coupled protein receptor 43 (GPR43), and β-actin (Table S2) were synthesized by Bioneer Inc. (Daejeon, Korea).

### Co-housing experiment

The co-housing experiment was performed for 7 days at the end of ABX treatment. Cecal contents and cecal tonsils were collected at 1/4(6 hours), 1, 3, 5, and 7 days after co-housing. Cecal contents were dissolved to 1 mg/ml. Dissolved cecal contents from control chickens and ABX-treated chickens were diluted 10–1,000 fold to adjust to the proper range of colony numbers (data not shown) and then spread on brain-heart infusion (BHI) agar media and incubated at 37 °C for 12 hours. CFU was determined by counting the number of colonies. All flow cytometric data were analyzed with FlowJo software.

### 16S rRNA sequencing of cecal microbiota

Genomic DNA was extracted from the ceca samples using the NucleoSpin Soil kit (MN, Düren, Germany) and used as a template in PCR amplification of the V4 region of the 16S rRNA gene with barcoded primer sets. The forward and reverse primers had common annealing sequences (5′-GTGCCAGCMGCCGCGGTAA-3′ and 5′-GGACTACHVGGGTWTCTAAT-3′), respectively, as used previously^64,65^. The PCR reaction was conducted with genomic DNA (5 ng), reaction buffer with 25 mM Mg^2+^ and 200 μM dNTP (each), 0.75 unit DNA polymerase (Ex-Taq, Takara, South Korea), and the barcoded primers (5 pmole each) under the following conditions: 94 °C for 3 min; 35 cycles of 45 s at 94 °C, 1 min at 55 °C and 90 s at 72 °C; and 72 °C for 10 min. Equal amounts of each PCR amplicon were pooled and further processed for construction of a sequencing library using the NEBNext® Ultra™ DNA Library Prep Kit (NEB, MA, USA). The library was sequenced with Illumina MiSeq to obtain 300-bp paired-end reads.

### Microbial community analysis

Paired Illumina reads were quality-filtered (≥Q20) and de-multiplexed using in-house Perl scripts^66^. The processed paired reads were merged into a single read for community analysis using the Quantitative Insights Into Microbial Ecology (QIIME) version 1.9.1. During QIIME analysis, selection of operational taxonomic units (OTU) was conducted based on a closed 16S rRNA database, Greengenes (gg_ptus-13_8-release version, 97% nucleotide identity). After OTU selection, we calculated the community diversity (α- and β-diversity) and relative abundance of each taxonomical group using QIIME. The number of observed OUTs was calculated using 2,000 reads assigned for OUT.

### Administration of short-chain fatty acids (SCFAs)

Upon hatching, chickens were fed a diet containing the SCFAs acetate (50 mM), butyrate (30 mM), and propionate (10 mM) (concentrations pre-determined, data not shown) for 7 days, and ABX was administered as a positive control.

### Measurement of concentration of SCFAs

Cecal contents were collected from chickens treated with ABX, and then centrifuged at 12,000 g. Supernatants were pooled and added with 200 μl of 25% meta-phosphoric acid. The concentration of SCFAs was measured by gas chromatography using an Agilent Tech 7890A (Hewlett Packard Strasse 876337, Waldbronn, Germany) of which a Supelco (30 m × 0.25 mm × 0.25 μm, fused silica capillary column) column was used.

### Statistical Analysis

Using SAS 9.3, statistical differences were determined using parametric or non-parametric t-test and one-way ANOVA with Turkey’s test. Differences were considered significant at *P* ≤ 0.05.

## Electronic supplementary material

Supplementary Information
