# Supplementary material for: Regulation of CD4+CD8−CD25+ and CD4+CD8+CD25+ T cells by gut microbiota in chicken
Source: Sci Rep. 2018 Jun 5;8:8627. doi: 10.1038/s41598-018-26763-0 (PMC5988814; doi:10.1038/s41598-018-26763-0)
Supplement: Supplementary file 1 — Supplementary Information [file 41598_2018_26763_MOESM1_ESM.docx]

**Regulation of CD4^+^CD8^-^CD25^+^ and CD4^+^CD8^+^CD25^+^ T cells by gut microbiota in chicken**

In Kyu Lee, Min Jeong Gu, Kwang Hyun Ko, Suhan Bae, Girak Kim, Gwi-Deuk Jin, Eun Bae Kim, Young-Yun Kong, Tae sub Park, Byung-Chul Park, Hyun Jung, Jung, Seung Hyun Han, and Cheol-Heui Yun

**Supplementary Results**

Supplementary Table 1. Elimination of gut microbiota in chickens treated with different concentrations of antibiotics in drinking water for 7 days.

| **DF** | **A** | **G** | **M** | **N** | **V** | **Unit** | **Elimination of microbes (%)** |
| --- | --- | --- | --- | --- | --- | --- | --- |
| 1:1 | 1 | 1 | 1 | 1 | 0.5 | g/L (mg/ml) | 99 > |
| 1:2 | 500 | 500 | 500 | 500 | 250 | mg/L (μg/ml) | 99 > |
| 1:10 | 100 | 100 | 100 | 100 | 50 |  | 99 > |
| 1:20 | 50 | 50 | 50 | 50 | 25 |  | 97 > |

* DF: Dilution factor, A: Ampicillin, G: Gentamycin, M: Metronidazole, N: Neomycin, V: Vancomycin


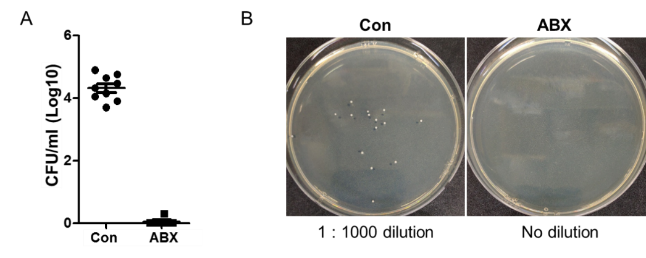


Supplementary Figure 1. Elimination of gut microbiota in chickens treated with antibiotics. Chickens were treated with distilled water (control, Con) or a mixture of antibiotics (ABX, 1:10) for 7 days. Ceca were collected from nine chickens, and cecal contents were plated on BHI agar plates with/without dilution and incubated for 12 hours at 37°C. (A) Colony forming units were determined by counting the number of colonies on the plate. Data are presented as the mean ± SD and are representative of three independent experiments. (B) One representative image from 10 similar results is shown.


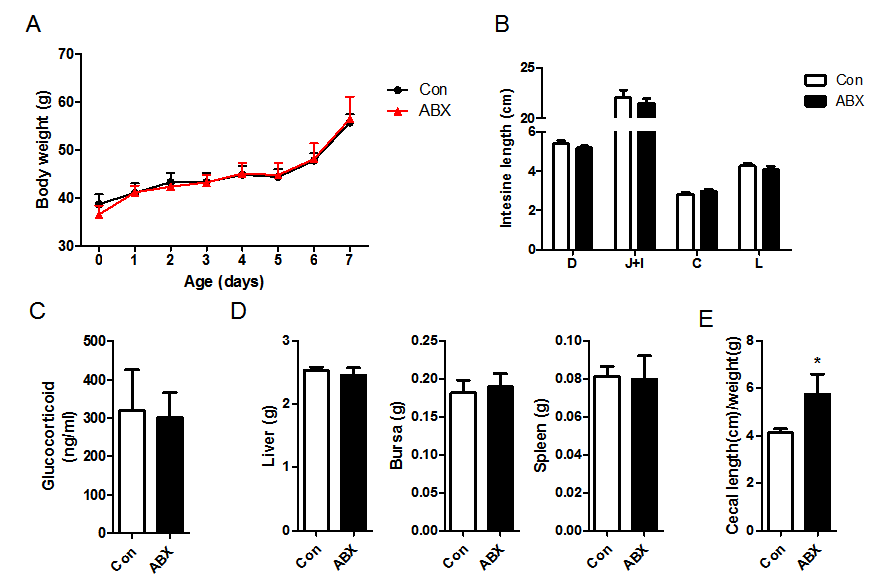


Supplementary Figure 2. Physiological changes in ABX-treated chickens. ABX was administered to chickens in drinking water from hatching for 7 days. (A) Body weight was measured daily. (B) Length of intestine (D: duodenum, J: jejunum, I: ileum, C: cecum, L: large intestine). (C) Amount of glucocorticoid by ELISA. (D) Weight of major immune organs. (E) Cecal length/weight. Data were obtained from six chickens in each group and are presented as the mean ± SD. **P*<0.05.

**Supplementary Figure 3. Gating strategy for CD4^+^CD8^–^CD25^+^ and CD4^+^CD8^+^CD25^+^ T cells.** Chickens at hatching were given water containing antibiotics for 7 days and cecal tonsils were taken. Single cells produced from cecal tonsils were, then, stained with anti-chicken TCRgδ, CD3, CD4, CD8a, and CD25 antibodies. CD3^+^TCRgδ^–^ cells gated were regarded as T cells, and then, CD4^+^CD8^–^CD25^+^ and CD4^+^CD8^+^CD25^+^ T cells are shown.


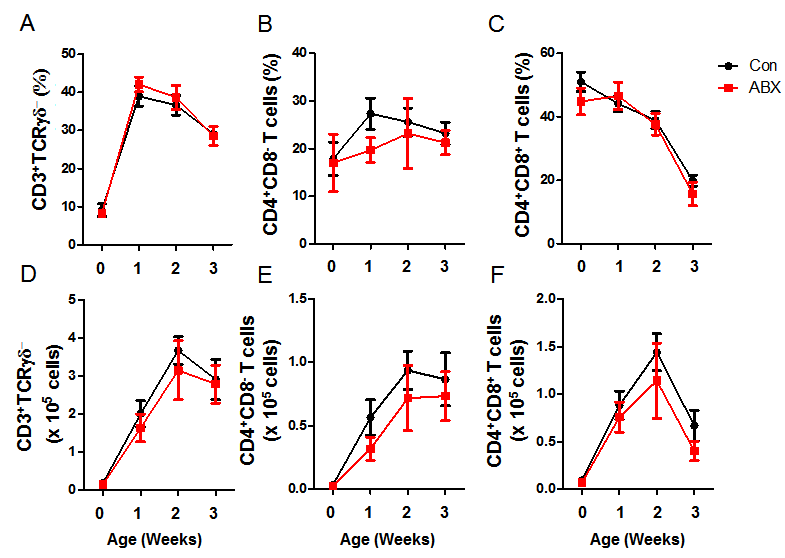


**0**

**1**

**2**

**3**

**0**

**1**

**2**

**3**

**Age (Weeks)**

**Supplementary Figure 4. Numbers of CD3^+^, CD4^+^CD8^–^ and CD4^+^CD8^+^ T cells were not changed in cecal tonsils from ABX-treated chickens.** Chickens were given water containing antibiotics at hatching for 3 weeks, and cecal tonsils were harvested. Single cells from cecal tonsils were stained with anti-chicken TCRγδ, CD3, CD4 and CD8α antibodies. The percentages of (A) CD3^+^γδTCR^–^, (B) CD4^+^CD8^–^, and (C) CD4^+^CD8^+^ T cells and the absolute numbers of (D) CD3^+^γδTCR^–^, (E) CD4^+^CD8^–^, and (F) CD4^+^CD8^+^ T cells are shown. (A-F) Data were obtained from six chickens in each group and presented as the mean ± SD.


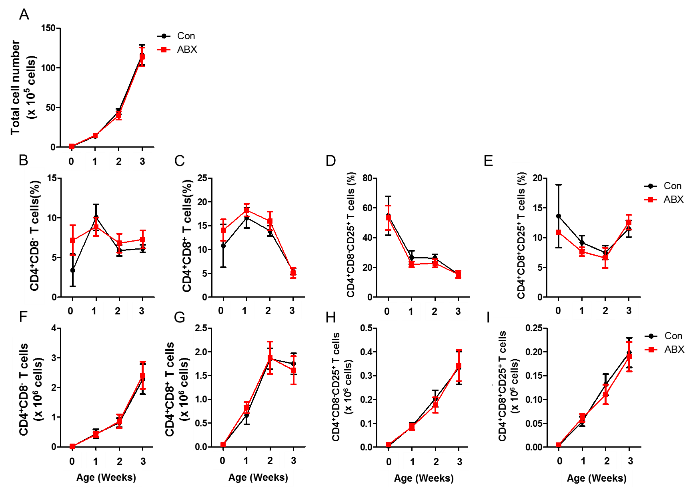


**Supplementary Figure 5. Levels of CD4^+^CD8^–^CD25^+^ and CD4^+^CD8^+^CD25^+^ T cells were not changed in spleen of ABX-treated chickens.** Chickens were given water containing antibiotics at hatching for 3 weeks, and the spleens were harvested. Single cells from each spleen were stained with anti-chicken CD4, CD8α, and CD25 antibodies. (A) Total number of cells and the percentages of (B) CD4^+^CD8^–^ T cells, (C) CD4^+^CD8^+^ T cells, (D) CD4^+^CD8^–^CD25^+^, and (E) CD4^+^CD8^+^CD25^+^ T cells and absolute numbers of (F) CD4^+^CD8^–^ T cells, (G) CD4^+^CD8^+^ T cells, (H) CD4^+^CD8^–^CD25^+^, and (I) CD4^+^CD8^+^CD25^+^ T cells are shown. (A-I) Data were obtained from six chickens in each group and presented as the mean ± SD.


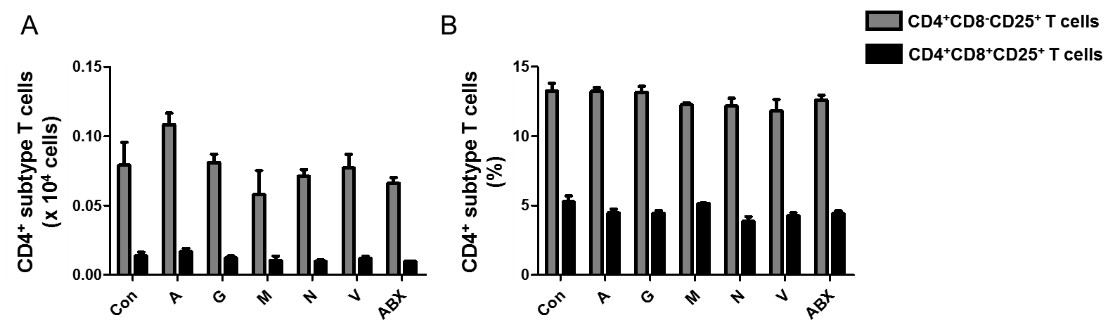


**Supplementary Figure 6. No changes in CD4^+^CD8^–^CD25^+^ and CD4^+^CD8^+^CD25^+^ T cells** **in chicken splenocytes after treatment with antibiotics.** Spleens were harvested from two -week-old chickens, and splenocytes were treated with a pre-determined concentration of each antibiotic or mixed antibiotics (ABX). (A) Cell numbers and (B) proportions of CD4^+^CD8^–^CD25^+^ and CD4^+^CD8^+^CD25^+^ T cells were examined by flow cytometry using anti-chicken CD4-FITC, CD8a-PE, and CD25-Alexa647 antibodies*.* Con, ABX non-treatment; A, ampicillin (100 μg/ml); G, gentamycin (100 μg/ml); M, metronidazole (100 μg/ml); N, neomycin (100 μg/ml); V; vancomycin (50 μg/ml); ABX, antibiotic cocktail as described in the Materials and Methods. (A and B) Data were obtained from three chickens and are representative of three independent experiments.


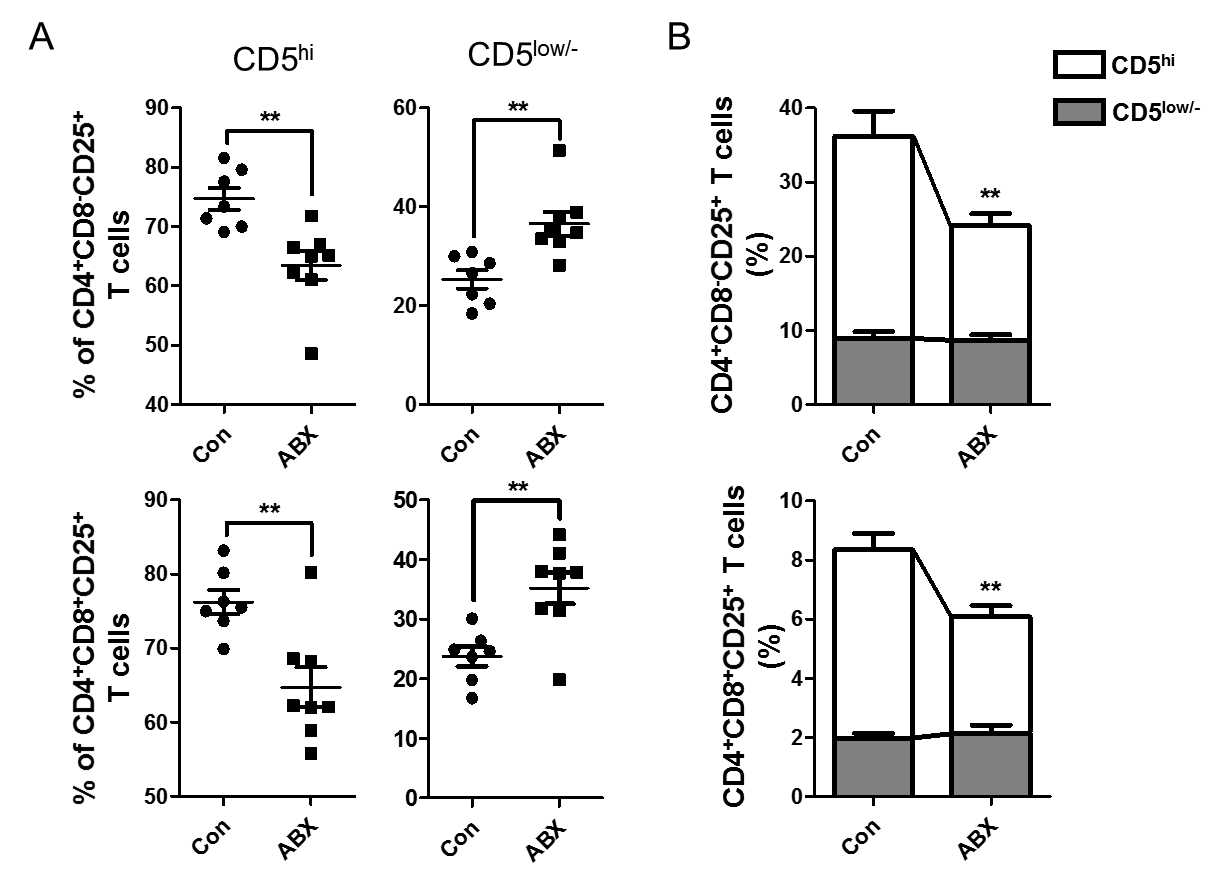


**Supplementary Figure 7. Reduction of CD5^hi^ cells in CD4^+^CD8^–^CD25^+^ and CD4^+^CD8^+^CD25^+^ T cells in ABX-treated chickens.** Eight chickens were given water containing antibiotics at hatching for 7 days, and cecal tonsils were harvested. Single cells from cecal tonsils were stained with anti-chicken CD4, CD5, CD8α, and CD25 antibodies. (A) The percentages of CD5^hi^ and CD5^low/–^ cells were analyzed in CD4^+^CD8^–^CD25^+^ and CD4^+^CD8^+^CD25^+^ T cells from cecal tonsils. (B) CD5^hi^ and CD5^low/-^ cells in CD4^+^CD8^–^ and CD4^+^CD8^+^CD25^+^ T cells were analyzed by using FlowJo. (A and B) Data were obtained from more than six chickens in each group and presented as the mean ± SD. Significant differences between Con and ABX are shown with asterisks. Data are representative of three independent experiments. ^**^*P*<0.01.


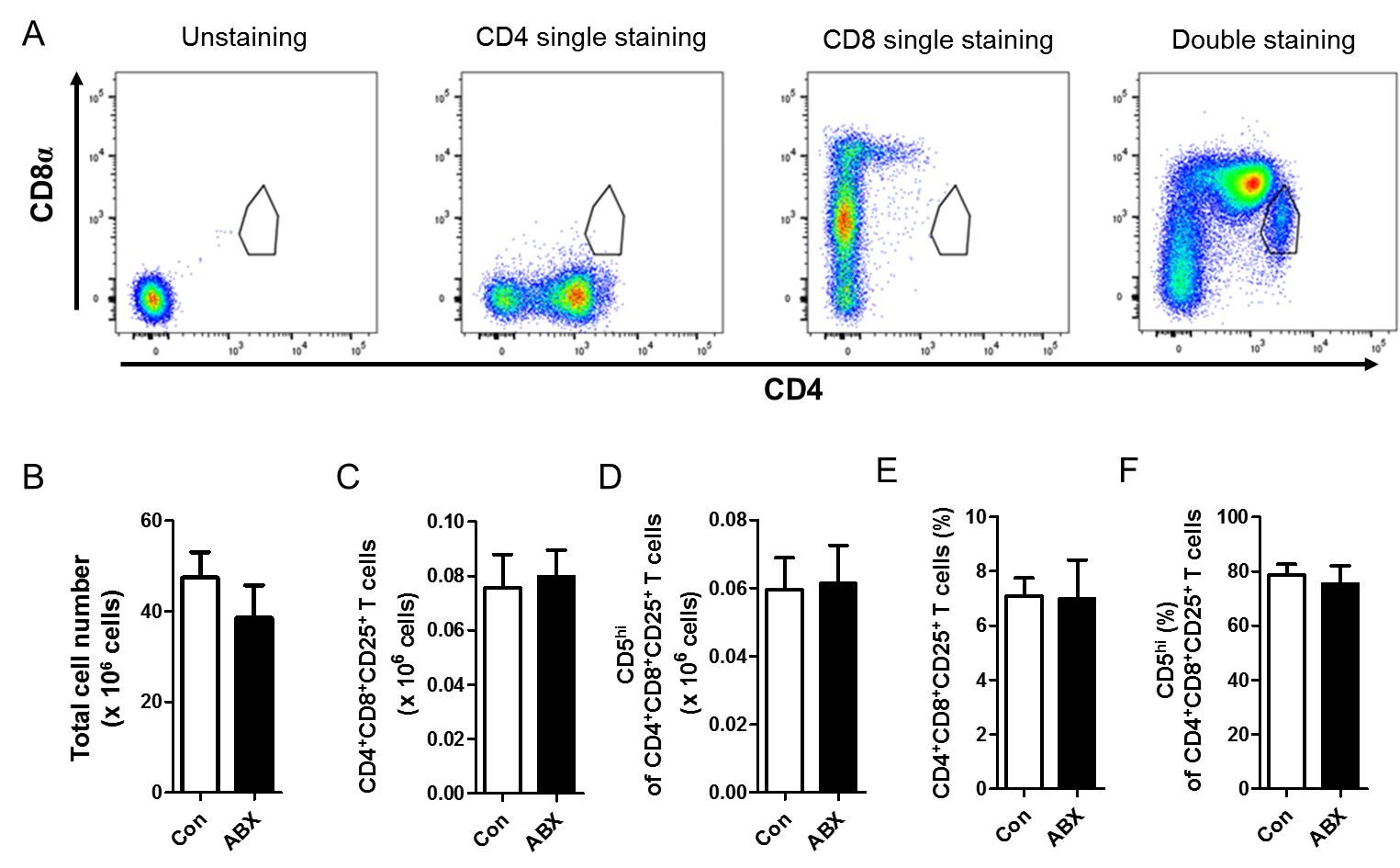


**Supplementary Figure 8. CD5^hi^ cells in the CD4^+^CD8^+^CD25^+^ subset of T cells were not changed in thymus of ABX-treated chickens.** Chickens were given water containing antibiotics at hatching for 7 days, and the thymus was harvested. Single cells from thymus were stained with anti-chicken CD4, CD8α, and CD25 antibodies. (A) Gating strategy was based on CD4 and CD8α expression. (B) Total cell number was obtained from a thymic lobe. Numbers of (C) CD4^+^CD8^+^CD25^+^ T cells and (D) CD5^hi^ of CD4^+^CD8^+^CD25^+^ T cells, and the percentages of (E) CD4^+^CD8^+^CD25^+^ T cells and (F) CD5^hi^ of CD4^+^CD8^+^CD25^+^ T cells are shown. (B-F) Data were obtained from six chickens in each group and presented as the mean ± SD.


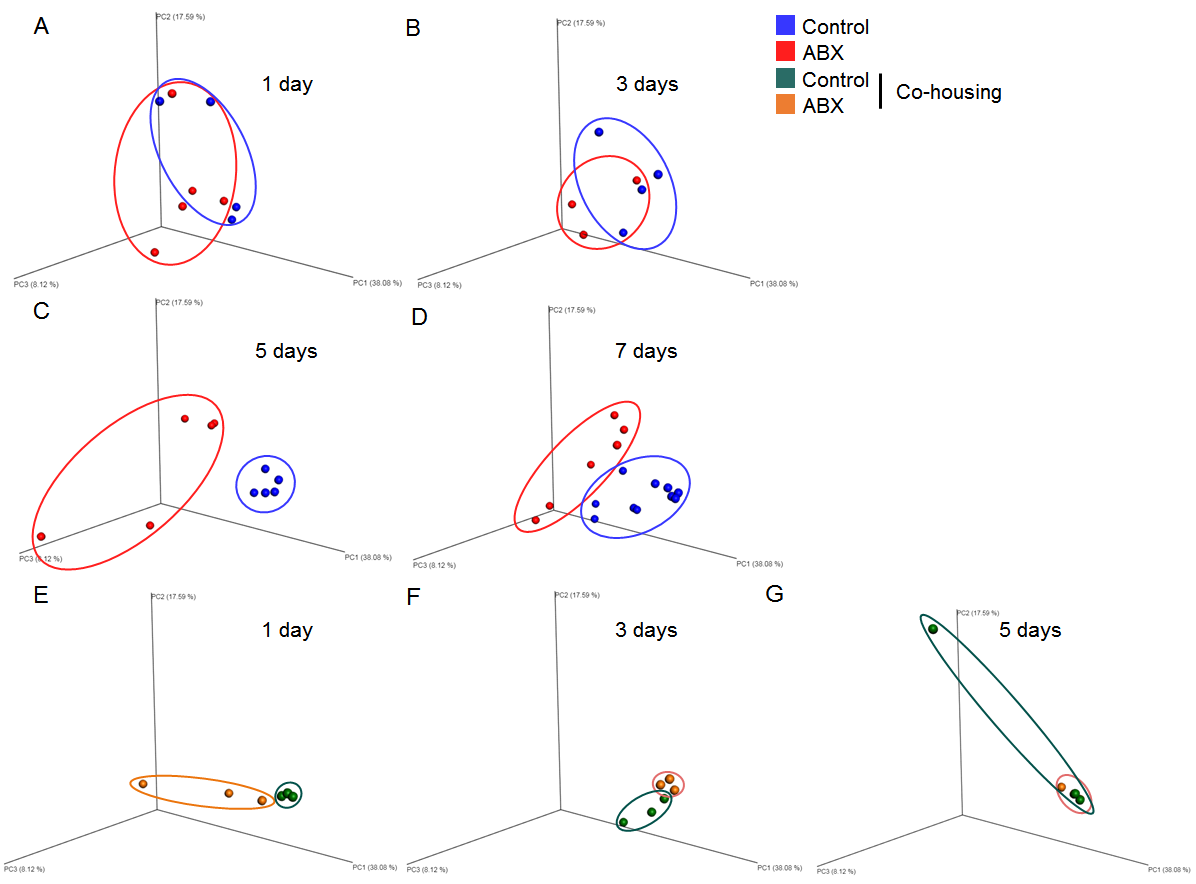


**Supplementary Figure 9. Principal coordinate analysis by weighted UniFrac for gut microbiota of chickens.** The beta diversity patterns were visualized in three-dimensional space. Each spot indicates an individual gut sample. All samples are shown on the same 3D space for comparison. Antibiotic-dependent shifts in gut microbiota were observed during the entire period. (A-D) During the antibiotics treatment period (ABX), gut microbial profiles of the two groups gradually separated and diverged. (E-G) After co-housing, however, gut microbial profiles gradually converged. PC1, PC2, and PC3 represent 38.08%, 17.59%, and 8.12% of the explained variance, respectively.

**
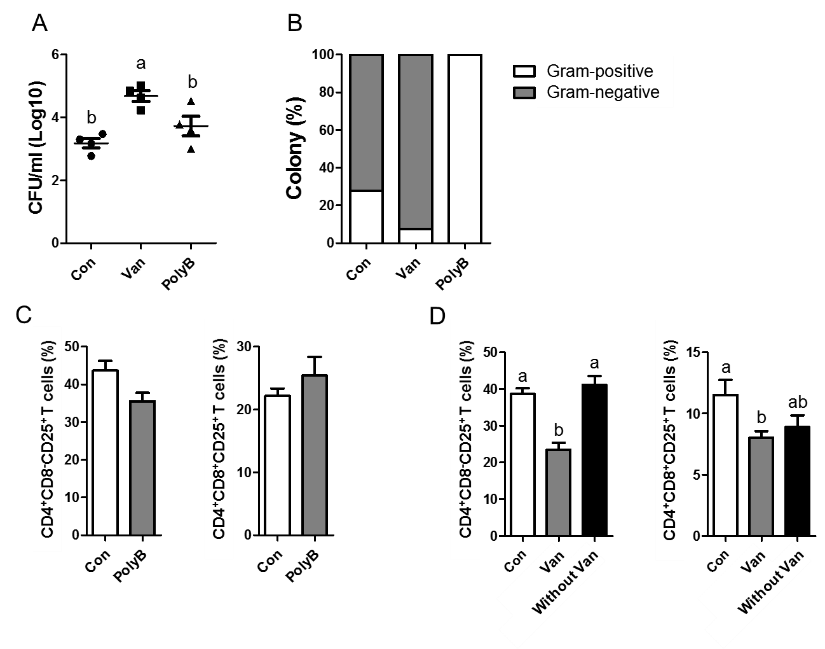
**

**Supplementary Figure 10. Elimination of Gram-positive bacteria was responsible for the changes in CD4^+^CD8^–^CD25^+^ and CD4^+^CD8^+^CD25^+^ T cells in ABX-treated chickens*.*** Chickens at hatching were treated with 50 mg/ml of vancomycin (Van), antibiotics without vancomycin (Without Van; ampicillin 100 mg/ml, gentamycin 100 mg/ml, metronidazole 100 mg/ml, neomycin 100 mg/ml), or polymyxin B (PolyB; 10 mg/ml) for 7 days and then co-housed with ABX-untreated control (Con) chickens for 7 days. (A) Colony forming units of cecal contents were measured from Van and PolyB groups, and (B) the composition of colonies was determined as Gram-positive or -negative colonies by Gram staining. (C and D) Proportions of CD4^+^CD8^–^CD25^+^ and CD4^+^CD8^+^CD25^+^ T cells in cecal tonsils were analyzed in chickens treated with vancomycin (Van), polymyxin B (PolyB), or antibiotics without vancomycin (Without Van) using flow cytometry and FlowJo. (A-D) Data were obtained from at least four chickens in each group and presented as the mean ± SD. Significant differences at *P* ≤ 0.05 are indicated by different letters.


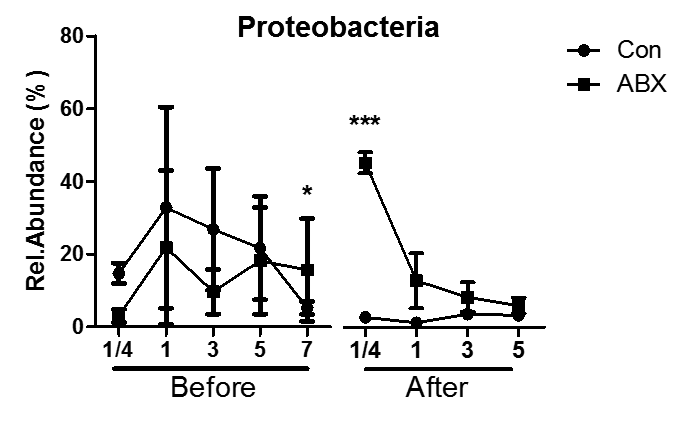


**Supplementary Figure 11. Abundance of Proteobacteria was increased in cecal contents from ABX-treated chickens after co-housing with control chickens.** Chickens were treated with ABX from hatching for 7 days and then co-housed with control chickens (Con) for 5 days. Relative abundance of 16S rRNA of Proteobacteria (Phylum) was determined by sequencing of cecal contents at 1/4 (6 hours), 1, 3, 5 and 7 days before, and 1/4 (6 hours), 1, 3 and 5 days after co-housing. Data were obtained from at least four chickens in each group and presented as the mean ± SD. Significant differences between Con and ABX are shown with asterisks at ^*^*P*<0.05 and ^***^*P*<0.001.


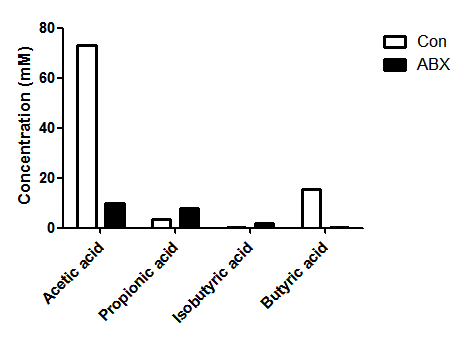


**Supplementary Figure 12. Concentration of SCFAs in the cecal contents in chickens treated with ABX.** Cecal contents of chickens treated with ABX were pooled. Concentration of SCFAs (acetic acid, propionic acid, isobutyric acid, butyric acid) was measured by gas chromatography.


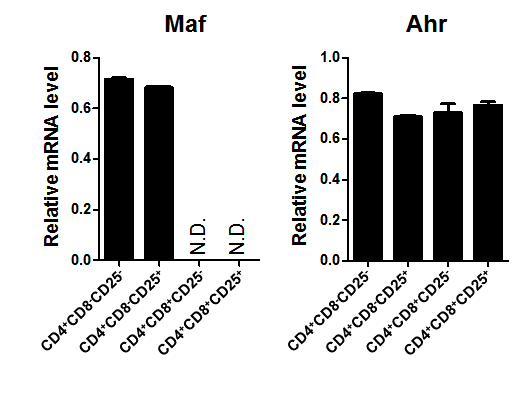


**Supplementary Figure 13. Maf and Ahr gene expression among CD4^+^ subtypes of T cells.** Single cells from cecal tonsils were stained with anti-chicken CD4, CD8a, and CD25 antibodies. Each subset of CD4^+^ T cells was sorted using an ARIA II FACS sorter. mRNA was extracted, and the levels of (A) Maf and (B) Ahr were determined by RT-qPCR. Data were obtained from three chickens and presented as the mean ± SD.

**Supplementary Table 2. List of primers used in the experiment**

| IL-10 | 3’-AGCTGACGGTGGACCTATTATT-5’  3’-GGCTTTGCGCTGGATTC-5’ | Forward  Reverse |
| --- | --- | --- |
| IFN-γ | 3’-CGGGAGCTGAGGGTGAA-5’  3’-GTGAAGAAGCGGTGACAGC-5’ | Forward  Reverse |
| Ahr | 3’- CAGGTCCCTGAAAACCTTGACT-5’  3’- ACGGCACCTGCATAACATGTT-5’ | Forward  Reverse |
| Maf | 3’- CCCCGTTACCTGAGGTCAGA-5’,  3’- GTCTTCGTGCCAGAACGTTGT-5’ | Forward  Reverse |
| G-coupled protein receptor 43  (GCR43) | 3’-CTCTTTATGGCTGCCCTCAG-5’  3’- GTAGCCCAGGCTTGGTTGG-5’) | Forward  Reverse |
| β-actin | 3’-CAACACAGTGCTGTCTGGTGGTA-5’  3’-ATCGTACTCCTGCTTGCTGATCC-5’ | Forward  Reverse |
